# Proposal of a Screening MIRU-VNTR Panel for the Preliminary Genotyping of *Mycobacterium bovis* in Mexico

**DOI:** 10.1155/2015/416479

**Published:** 2015-04-05

**Authors:** Enrique Bolado-Martínez, Iliana Benavides-Dávila, Maria del Carmen Candia-Plata, Moisés Navarro-Navarro, Magali Avilés-Acosta, Gerardo Álvarez-Hernández

**Affiliations:** ^1^Departamento de Ciencias Químico Biológicas, Universidad de Sonora, Boulevard Luis Encinas y Rosales, 83000 Hermosillo, SON, Mexico; ^2^Departamento de Medicina y Ciencias de la Salud, Universidad de Sonora, Boulevard Luis Donaldo Colosio y Francisco Q. Salazar S/N, 83000 Hermosillo, SON, Mexico; ^3^Laboratorio Estatal de Salud Pública, José Miró Abella S/N, 83294 Hermosillo, SON, Mexico

## Abstract

*Mycobacterium bovis* is the major causative agent of bovine tuberculosis, one of the most relevant zoonoses in the world, and affects a wide range of wild and domesticated animals. Development of screening panels in mycobacterial genotyping, according to specific geographical regions, is strongly needed. The aim of this study is to select a panel, constituted by highly polymorphic MIRU-VNTR *loci*, to discriminate clinical isolates of *M. bovis* in Mexico. In this study, 65 isolates of *M. bovis* obtained from clinical bovine samples proceeding from different geographic regions of Mexico were identified by phenotypic and genotypic tests and subsequently genotyped by a 24-*locus* MIRU-VNTR panel. The most polymorphic *loci* were selected to build a panel with a high discriminatory power similar to the 24-*locus* panel results. A panel of seven elements (QUB 11a, MIRU 26, ETR-A, QUB 26, MIRU 16, MIRU 27, and MIRU 39) with the highest allelic diversity showed an appropriate differentiation. The selected MIRU-VNTR elements, according to the regional allelic variability, may be used in the preliminary genotyping of *Mycobacterium bovis* isolates in Mexico.

## 1. Introduction

*Mycobacterium bovis* is the major causative agent of bovine tuberculosis (BTB), one of the most relevant zoonoses in the world, showing a wide range of effects on wild and domesticated animals [1–3]. BTB is a major worldwide animal health problem, which negatively affects animal production as well as national and international trade of livestock [4]. In developed countries, which have a tradition of cattle farming, the prevalence of BTB has reached very low levels because of strict control policies [5], but even in the United States of America* M. bovis* continues infecting cattle at a low level despite control and eradication efforts [6]. Currently, only three countries, Canada, Mexico, and Australia, are authorized to trade live cattle with the United States [6]. However, BTB precludes animal-related trade and production, causing financial losses to farming worldwide, and remains a public health hazard [1].

There is limited evidence of human-to-human transmission of BTB. In fact, recent research findings suggest that transmission occurs mainly within cattle populations and less frequently between cattle and humans or between humans [7]. The geographical distribution of* M. bovis* cases in more-rural areas is consistent with zoonotic transmission [8]. However, the accurate incidence of BTB may be underestimated given that the infection is clinically indistinguishable from that caused by* M. tuberculosis* and because it is difficult to differentiate* M. tuberculosis* from* M. bovis* with traditional diagnostic methods [9, 10].

Furthermore, for BTB control it is required to accurately know how many and which* M. bovis* strains are located in each geographic region, and classical bacteriological methods are unable to distinguish strains among the same species [2]. Strain genotyping may contribute to a better understanding of BTB transmission and could lead to identification of outbreaks and tracking dissemination of particular strains, to explore relations between domestic and wild BTB, to improve bovine TB control strategies, or even to reconstruct the evolution of a certain group of bacteria [3, 5, 11, 12].

There is a growing research showing that mycobacterial interspersed repetitive unit variable number tandem repeat (MIRU-VNTR) is the most discriminatory technique to genotype* M. bovis* isolates [1, 13, 14]. This method can therefore be used to confirm conventional epidemiological links, trace transmission routes, determine the source of infection and outbreaks, understand the relationship between different outbreaks, and identify wild animal reservoir of* M. bovis* [5, 8].

In Europe, a network of laboratories has agreed on a consensus of six VNTR* loci* to genotype* M. bovis* [12]; however, these* loci* are still not widely used, which can hamper interlaboratory exchange [11, 13]. In Mexico, where resources for diagnosis and molecular typing are scarce, development of screening panels in mycobacterial genotyping is strongly needed. The aim of this study was to select a minimal MIRU-VNTR* loci* panel to discriminate locally prevalent* M. bovis* population.

## 2. Materials and Methods

### 2.1. Clinical Isolates

**Figure 1:**
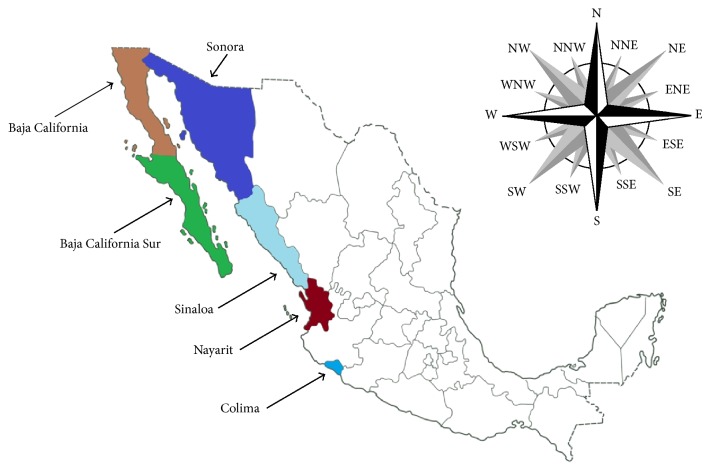
Map of Mexico and geographical origin of the 65* Mycobacterium bovis* isolates included in this study.

Sixty-five* M. bovis* isolates, originally cultured from bovine tuberculosis lesions, were included in this study. The isolates were obtained from five states of Mexico (Figure 1), Baja California (11 isolates), Baja California Sur (5), Colima (12), Nayarit (12), Sinaloa (12), and Sonora (13), from 2010 to 2013 and sent to a reference laboratory (Laboratorio Estatal de Salud Pública) in Sonora, México. All isolates were identified as* M. bovis* by phenotypic (standard biochemical assays) [15] and genotypic (*gyrB*-RFLP and RD1) tests [16, 17]. All isolates were frozen in skim milk and kept at −20°C until use.* Mycobacterium tuberculosis* H37Rv strain was used as control.

### 2.2. DNA Preparation

*M. bovis* isolates and* Mycobacterium tuberculosis* H37Rv strain frozen in skim milk were thawed and cultured on Stonebrink or Löwenstein-Jensen media, respectively, prepared as solid slants in screw-cap tubes, for 3 to 4 weeks at 37°C. For each isolate, four to five colonies were transferred into 500 *μ*L of TE buffer (0.01 M Tris-HCl, 0.01 M EDTA [pH 8.0]). The suspended colonies were washed twice with TE and boiled for 30 min in Chelex-100 (10% in MilliQ water), and the bacterial lysate was used directly in PCRs (modified from [18, 19]).

### 2.3. MIRU-VNTR

Twenty-four genomic* loci* were amplified in separate PCR reactions with the primers previously described [20, 21]. PCRs were performed on 2.5 *μ*L of DNA sample in a final volume of 25 *μ*L. All PCR reactions were carried out according to the method of Supply et al. [22] with the following modifications: the final MgCl_2_ concentration was 1.5 mM, and 0.5 U of TaqDNA polymerase (Promega) was used in each reaction. Additionally, QUB 26* loci* required 0.05% (v/v) DMSO for optimal results. All MIRU-VNTR* loci* were amplified in individual reactions. The amplification program consisted of 2 min at 95°C, followed by 40 cycles of 60 s at 94°C, 60 s at 59°C, and 90 s at 72°C and a final extension at 72°C for 10 min. The number of tandem repeats (alleles) was estimated after electrophoresis on 2% agarose (Sigma) gels at 90 V with a 100 bp ladder (Promega) according to the allele calling table.* Mycobacterium tuberculosis* H37Rv strain was used as positive control in every PCR reaction and electrophoresis procedure.

### 2.4. Data Analysis

The Hunter-Gaston index (HGI) was calculated to determine the discriminatory power for individual VNTR* loci* [23]. MIRU-VNTR profiles were recorded as character data and analyzed using Bionumerics software v 6.6 (Applied Maths, St-Martin-Latem, Belgium). Dendrograms were generated by using the categorical character option and the UPGMA (for unweighted pair-group method with arithmetic averages) clustering method.

## 3. Results

**Figure 2:**
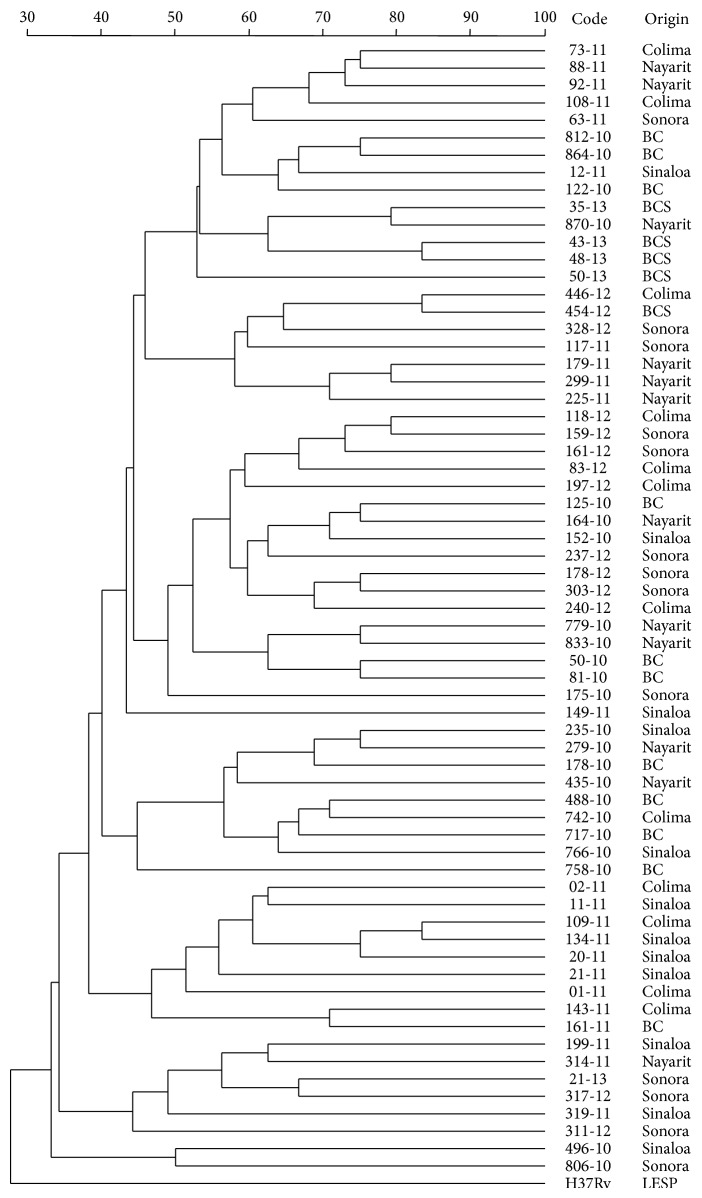
Dendrogram generated by using categorical character and the UPGMA clustering method with 24-*locus* MIRU-VNTR panel results of 65 isolates of* M. bovis* and* M. tuberculosis* strain H37Rv.

As expected, the 24-*locus* MIRU-VNTR panel allowed differentiation of 65 isolates.  Figure 2 shows that each* M. bovis* isolate has a unique MIRU-VNTR profile and H37Rv* M. tuberculosis* strain is quite different from the rest of the mycobacteria. Despite achieving individual differentiation of each microorganism evaluated, some kind of association, regarding the geographical origin of each isolate, was not possible to obtain.

The Hunter-Gaston index differed for individual* loci*, ranging from 0.34 to 0.86 (Table 1).* Loci* QUB3232 and QUB11a were the most discriminant, whereas QUB 1895 and MIRU 20 had the lowest diversity index.* Locus* QUB3232 showed several problems and was subsequently excluded from the final selection of highly discriminative MIRU-VNTR* loci*.

Once the most polymorphic MIRU-VNTR* loci* were identified, additional analysis was performed to identify combinations for improved differentiation of mycobacteria included in the present study.

**Figure 3:**
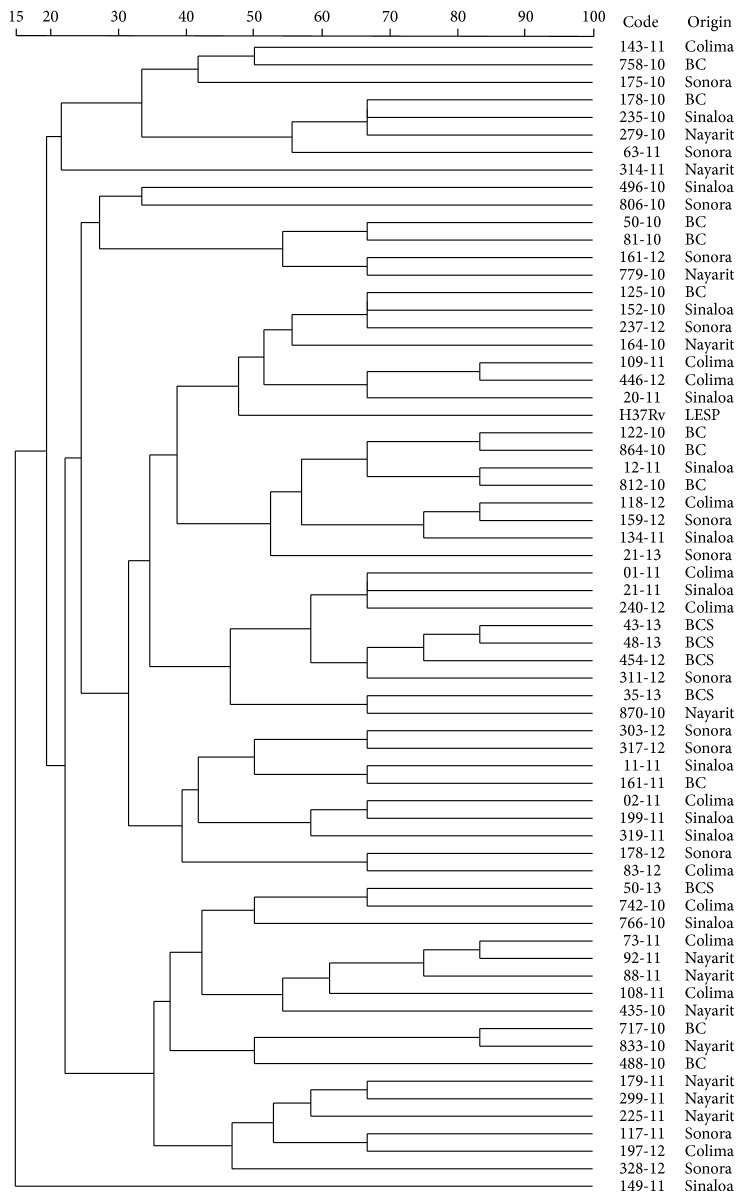
Dendrogram generated by using categorical character and the UPGMA clustering method with 7-*locus* MIRU-VNTR panel results of 65 isolates of* M. bovis* and* M. tuberculosis* strain H37Rv.

In the first approach, five MIRU-VNTR typing* loci* (excluding QUB 3232) showed a very high discriminatory index for our set of isolates and were particularly useful to differentiate almost all the mycobacterial isolates, except 73-11 and 92-11 isolates that displayed an identical profile (data not shown). By including MIRU 27, differentiation of each of the isolations was achieved; however, when we incorporated MIRU 39 into the panel (seven-*locus* MIRU-VNTR panel), the differentiation was kept and additionally it was possible to observe some clusters, according to the geographical region of origin (Figure 3).

## 4. Discussion

The value of mycobacterial interspersed repetitive units variable number tandem repeats (MIRU-VNTR) as a genotyping technique for* Mycobacterium bovis* has previously been confirmed [1]. However, a standardized panel of MIRU-VNTR* loci* has not yet been adopted for* M. bovis*, since allelic diversity of each* locus* differs among countries [1, 4, 11]. Thus, it is important to standardize a panel of* loci* for future interlaboratory comparisons [4].

The first study in Mexico where genetic diversity of mycobacterial strains was evaluated using MIRU-VNTR revealed seven distinct patterns in nine* M. bovis* strains [10]. Recently Laniado-Laborín and colleagues found 26 strains of* M. bovis* in 600 clinical mycobacterial isolates [9]. In both studies, however, the mycobacteria were recovered from human clinical samples. In this study we have identified 65* M. bovis* isolates with unique MIRU-VNTR patterns, but all of them were recovered from bovine sources.

Although all the 24* loci* MIRU-VNTR showed good discriminatory power, the* loci* with the highest diversity were QUB 3232, QUB 11a, MIRU 26, ETR A, QUB 26, MIRU 16, MIRU 27, MIRU 39, MIRU 2, MIRU 31, and QUB 3336 (HGI 0.85–0.60). Meanwhile, the* loci* with intermediate diversity were QUB 23, ETR C, QUB 11b, ETR B, MIRU 40, MIRU 23, QUB 18, MIRU 10, MIRU 4, MIRU 24, and QUB 15 (HGI 0.58–0.42). Finally* loci* QUB 1895 (HGI: 0.38) and MIRU 20 (HGI: 0.34) were considered with low discriminatory power.

Our findings are consistent with data reported by previous researchers that consider QUB 3232 as the most variable* locus* [1, 4, 11, 14, 24]. QUB 11a, the second most variable* locus* in this study, has been reported with high [1, 7, 11, 13, 14, 20], intermediate [24], or low diversity index [4]. This situation was detected for MIRU 26 (high [1, 3, 7, 9, 20], intermediate [14, 24], and low diversity index [2]) and QUB 26 (high [7, 11, 20] and intermediate diversity index [2, 14, 24]).

Our results are also consistent with previous studies where ETR A was considered as a highly polymorphic* locus* [1, 4, 7, 11, 14, 20, 24]. However we observed considerable differences in utility of the* loci* MIRU 2, 27, 31 and 39, which in previous studies were considered as having a discriminatory power of intermediate or scarce value [3, 24]. Interestingly, in a previous research, MIRU 31 (HGI = 0.61, in this study) showed null discriminatory power in the genotyping of* M. bovis* recovered from human samples in Mexican population [9].

Regarding the* loci* that showed an intermediate or low value of HGI, our results correlate more appropriately with previous studies [1–4, 11, 13, 14, 20, 24].

By testing combinations of the most variable elements, within the set of 24* loci*, we found that a satisfactory degree of discrimination could be achieved using a minimal group of six* loci*; however, we recommend extending the set of markers to include MIRU 39 (7-*locus* MIRU-VNTR panel), since it is possible to observe some tendency to clustering, according to the geographical region of origin (Figure 3). Several attempts to reduce the number of repetitive elements and maintain the most discriminative power have been done, and that includes six [4], eight [1], nine [20], or even thirteen MIRU-VNTR* loci* panels [24]. However, the optimal procedure to use for strain typing of* M. bovis* will depend on the strains present in a region, the number of isolates to be typed, and availability of resources.

As already mentioned, even though QUB 3232 was the most discriminative* locus*, difficulties with the reproducibility of amplification and gel band previously reported [9, 20, 24] lead us to not recommending this* locus* for routine use.

This is a preliminary screening investigation to establish a minimal panel for the large-scale genotyping analysis of* M. bovis* in Mexico. Our findings suggest the MIRU-VNTR markers that have high discriminative power and may be used to identify* M. bovis* isolates from the northwest and probably all of the Mexican territory. However, further research is recommended, to determine MIRU-VNTR* loci* that would be sufficiently discriminating in different settings/profiles of circulating strains, as previously proposed [8, 12].

## 5. Conclusions

The present study demonstrated the usefulness of 24-*locus* MIRU-VNTR panel for discriminating 65* Mycobacterium bovis* isolates from six different regions of Mexico. This study also presents seven-*locus* MIRU-VNTR panel with high discriminatory power. Additional studies are needed to validate the proposed scheme.

## Figures and Tables

**Table 1:** Allelic diversity of 24-*locus* MIRU-VNTR in 65 *Mycobacterium bovis* Mexican isolates.

*Locus *	Number of copies														HGI
	1	2	3	4	5	6	7	8	9	10	11	12	13	14	
QUB 3232					4	3		6	15	14	9	6	4	4	0.86
QUB 11a				2	4	1	4	2	11	20	16	5			0.81
MIRU 26	1		1	7	14	21	14	5	2						0.80
ETR A				1	4	2	14	22	18	4					0.77
QUB 26		1	23	27	10	4									0.69
MIRU 16	24	28	10	3											0.66
MIRU 27		4	37	10	10		1	3							0.63
MIRU 39	2	36	14	10	1	2									0.63
MIRU 2	10	34	20	1											0.62
MIRU 31	1	23	33	8											0.61
QUB 3336	1	40	4	3	6	8	1		2						0.60
QUB 23				16	39	7	2	1							0.58
ETR C	3	8	12	41	1										0.56
QUB 11b	2	4	6	41	12										0.56
ETR B		1	2	40	21	1									0.52
MIRU 40	18	42	5												0.51
MIRU 23		1	24	40											0.49
QUB 18		9	45	9	2										0.49
MIRU 10	17	44	4												0.48
MIRU 4			43	22											0.45
MIRU 24	22	43													0.45
QUB 15	3	48	13	1											0.42
QUB 1895		12	50	3											0.38
MIRU 20	8	52	5												0.34

HGI: Hunter-Gaston index.
